# Ticks home in on body heat: A new understanding of Haller’s organ and repellent action

**DOI:** 10.1371/journal.pone.0221659

**Published:** 2019-08-23

**Authors:** Ann L. Carr, Vincent L. Salgado

**Affiliations:** BASF Corporation, Research Triangle Park, North Carolina, United States of America; University of Kentucky College of Medicine, UNITED STATES

## Abstract

Ticks are second only to mosquitoes as vectors of disease to humans and animals. Tick host detection is mainly ascribed to Haller’s organ, a complex sensory structure on the tick foreleg that detects odors, carbon dioxide and heat, but these host detection mechanisms are not well understood. There is anecdotal evidence that ticks and other ectoparasites are attracted to heat, but it has never been demonstrated that they use radiant heat to detect hosts at a distance. In fact, previous attempts to do this have concluded that radiant heat was not used by ticks. Here we use a novel thermotaxis assay to investigate the detection range, temperature dependence and repellent sensitivity of heat perception in ticks and to identify the sensory organ responsible for this sense. We show that *Amblyomma americanum* and *Dermacentor variabilis* ticks can locate a human from several meters away by radiant heat sensed by the part of Haller’s organ known as the capsule, a covered spherical pit organ. An aperture in the capsule cover confers directionality and highly reflective interior surfaces of the capsule concentrate radiation on the sensilla to sharpen directionality and increase sensitivity. Commercial insect repellents provide an effective means of personal protection against potentially infectious tick bites by hindering host-seeking behavior. Low concentrations of the insect repellents DEET, picaridin, 2-undecanone, citronellal and nootkatone eliminate thermotaxis without affecting olfaction-stimulated host-seeking behavior. Our results demonstrate that the tick Haller’s organ capsule is a radiant heat sensor used in host-finding and that repellents disrupt this sense at concentrations that do not disrupt olfaction. We anticipate that this discovery will significantly aid insect repellent research and provide novel targets for the development of innovative integrated pest management programs and personal protection strategies for ectoparasites and vector-borne disease.

## Introduction

Ticks transmit the widest array of pathogens of any arthropod vector, including viruses, bacteria, fungi, protozoa and helminths, and are second in importance only to mosquitoes as vectors of disease to humans and animals[1]. The American dog tick *Dermacentor variabilis* (Acari: Ixodidae) and the lone star tick *Amblyomma americanum* (Acari: Ixodidae) are the two most abundant ticks in the US [2]; and both have the capacity to transmit ehrlichiosis, tularemia and Rocky Mountain spotted fever. Also of public health concern is the increasingly prevalent galactose-alpha-1,3-galactose red meat allergy associated with *A*. *americanum* bites [2, 3]. Tick bites and host blood feeding are the primary routes of pathogen exposure and a key determining factor of tick vectoral competence.

Host-seeking is essential for tick viability, with host bloodmeals required for tick development and reproductive maturation. *A*. *americanum* and *D*. *variabilis* are non-nidiculous tick species utilizing two host-seeking strategies throughout their life cycles: active host-hunting and vantage point host-ambushing. Both strategies involve multiple sensory modalities, though olfaction is believed to be the predominant sensory modality guiding tick host-seeking [4–6]. Tick olfaction is ascribed mainly to Haller’s organ, a localization of chemosensory sensilla on the tarsus of the foreleg. While both components of Haller’s organ, a posterior capsule and anterior pit, are thought to serve olfactory functions, there is morphological evidence that supports putative hygrosensory and/or thermosensory functions [4, 6–8]. Of particular interest to us was the physical organization and structure of the posterior capsule, a sensilla-containing spherical pit with a cuticular cover perforated by an aperture [9, 10]. We postulate that these physical attributes make the posterior capsule better suited for the detection of radiation than odorants: while the aperture would severely hinder odorant diffusion, it would confer directionality for radiation-sensing by restricting the field of view. In fact, the posterior capsule structure is strikingly camera-lucida-like, suggesting that it could be a very sensitive detector for thermal infrared radiation (IR), which would be of value in sensing hosts at a distance, by their radiated heat.

Unfortunately, the only study to investigate the role of radiant heat in tick host-seeking concluded that ticks were not attracted to radiant heat, and that larval and nymphal ticks attracted to the heat source in that study, a 37 °C glass tube, were responding to only a temperature gradient [7]. However, the methods used in that study to distinguish between radiant and convective heat were flawed, as we show in the methods section of this paper. Recently, it was shown that ticks were attracted to infrared light with a wavelength of 880 nm, but not after amputation of the foretarsae, including the Haller’s Organs [11]. The relevance of this finding for heat perception is not clear, since the wavelength of the infrared radiation used was well outside the range of thermal IR emitted by human skin, which includes wavelengths between 5 and 20 μm, with a peak near 10 μm [12].

We show here that the Haller’s organ capsule is indeed an exquisitely sensitive radiant heat sensor that guides host-finding. Our results show that female and male *A*. *americanum* and *D*. *variabilis* ticks thermotax from distances of at least 1 m toward a 10 X 10 cm high-emissivity surface warmed to a temperature of 37 °C, but not when that surface was covered with aluminum foil to make it a poor emitter of thermal infrared radiation. The distance sensitivity of thermotaxis indicates that female *A*. *americanum* could detect the body heat from an adult human at a distance of up to 4 meters under ideal conditions. Occlusion of the Haller’s organ capsule aperture with wax eliminated thermotaxis, confirming that the capsule is a directional sensor for thermal IR. We further show that exposure of ticks to the common repellents DEET, picaridin, 2-undecanone, citronellal and nootkatone disrupted thermotaxis at concentrations of 50 ng•cm^-3^, whereas DEET at twice this concentration did not affect the olfactory attraction to CO_2_, a known host-seeking stimulant of ticks. These results indicate that repellents specifically disrupt the IR sense of ticks.

## Materials and methods

### Ticks

Unfed virgin adult female and male *Amblyomma americanum* (lone star tick) and *Dermacentor variabilis* (American dog tick), two weeks post-molt, were purchased from Ecto Services Inc. (Henderson, NC, USA). Both colonies were started with field-collected *A*. *americanum* and *D*. *variabilis* obtained from a single site in Stillwater, OK and maintained with subsequent collections from the same site. Adult female and male *A*. *americanum* and *D*. *variabilis* were maintained in separate containers at 23 ± 1 °C and 97% R.H., with a short-day photoperiod of 10 h light: 14 h dark, including 1h-long dusk and dawn periods under red light at the beginning and end of each scotophase. Short days were used because of the observation that *Dermacentor* and *Amblyomma* ticks become more active during late summer and early fall, when daylight decreases to 10–11 hrs [2, 3]. While the effect of short days has not been confirmed with ticks in laboratory studies, short days have been found to increase host-seeking, feeding and longevity of laboratory-reared adult *Aedes albopictus* and *A*. *aegypti* [13].

### Thermotaxis assays

We used a thermotaxis assay to investigate detection range, temperature dependence and repellent sensitivity of heat perception in ticks and to identify the sensory organ responsible for this sense. A warm surface rather than an electronic infrared radiation (IR) source was used as the target for thermotaxis, since according to Planck’s Law the radiant heat emitted by the warm surface would closely mimic the spectrum and intensity of thermal radiation emitted by human skin, which has an emissivity of at least 0.98 [12], allowing the determination of the temperature sensitivity of the radiant heat sense. Furthermore, it is important to use a large enough surface as a radiant heat source because, while the intensity of radiation from each point on the surface decreases as the square of distance, the surface area within the field of view of the radiant heat sensor increases by the same factor, so that the intensity of radiant heat entering the sensor is independent of distance as long as the target fills the entire field of view. The key parameters of a radiant heat sensor are therefore temperature sensitivity and field of view.

**Fig 1:**
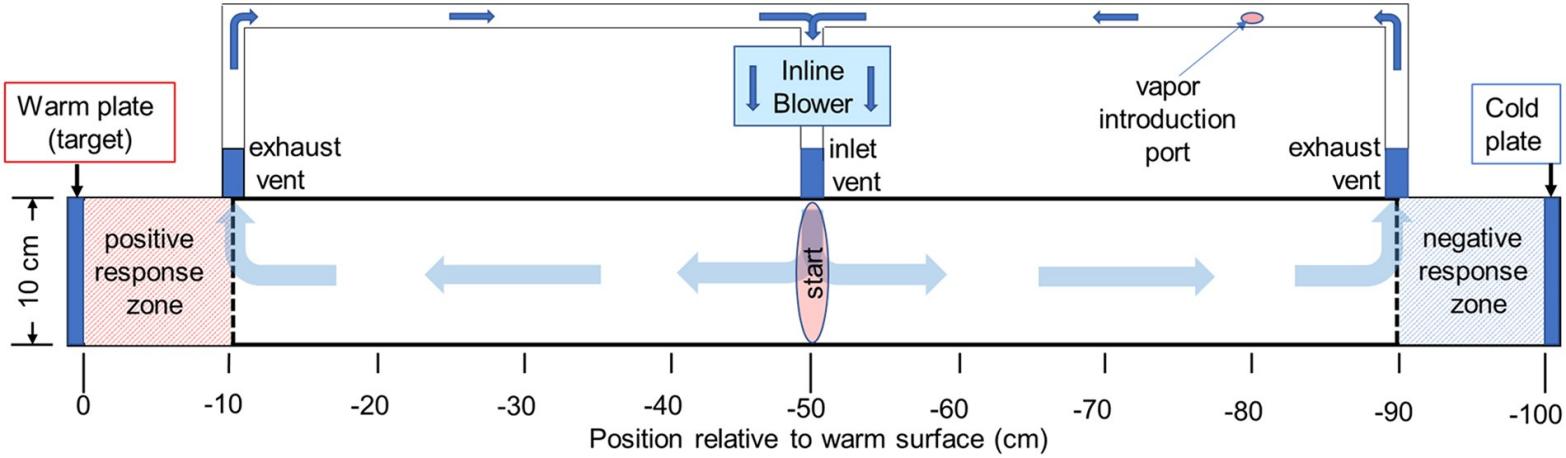
Thermotaxis bioassay arena with closed air circulation system.

Thermotaxis assays were conducted in an arena consisting of a long box with interior dimensions of 10 cm x 10 cm x 100 cm (Fig 1). The long walls and floor of the box were made of ¾ inch birch plywood painted with flat white latex paint, and the top was a tight-fitting removable lid made from ¼ inch thick Plexiglas to allow observation from above while blocking thermal radiation from outside the arena. Each end wall of the arena was the 10 cm x 10 cm blue anodized aluminum surface of a temperature-controlled thermoelectric cold plate (TEC plate, model TCP-50, Advanced Thermoelectric, Nashua, NH, USA), controlled by a TEC controller (model 1000–0649) and WinVue software v.4.011 from Vuemetrix (San Jose, CA, USA). All parts of the arena fit tightly enough that ticks could not escape. The arena was always at room temperature (22 ±1 °C), while the TEC plates presented temperature-controlled surfaces with an emissivity of 0.94 (measured with a FLIR E8 thermal imaging camera, FLIR Instruments Inc., Wilsonville, OR, USA)) at either end of the arena. All TEC plate temperatures were verified with a precision thermocouple thermometer (BAT-12, Physitemp Instruments Inc., Clifton, New Jersey, USA), with the thermocouple inserted into the center of the 1 cm-thick aluminum TEC plate through a hole drilled into one side and filled with heat-conducting paste. The controller’s feedback thermistor was in the same location. The thermal imaging camera was used to verify arena temperatures and to check for unintended infrared reflections. We specifically ensured that reflection of thermal radiation from the warm plate at 50 °C in the cold plate at RT was undetectable, as such reflections would confuse the ticks and decrease the thermotaxis toward the warm plate.

Thermotaxis trials were conducted during the day between the hours of 10:00–16:00 at room temperature (RT, 22 ± 1°C), with a relative humidity of 40%, under LED lighting as described below. One TEC plate, designated the cold plate or end wall, was programmed to present a RT surface, and the opposing TEC plate, designated the target, was programmed to present various temperatures between RT and 50°C. Control trials with both TEC plates set to RT were performed to document the normal behavior of ticks in the test arena and to ensure that there were no biases associated with the laboratory conditions or experimental set-up. Unless stated otherwise, a thermotaxis trial consisted of six runs of eight ticks. Ticks were randomly selected from a total population of 250 and acclimated to test conditions in vials next to the arena for 20 min prior to use. Ticks were released on the floor at the center of the arena (-50 cm) and their movements were filmed for 5 min, unless noted otherwise. To eliminate contamination and positional bias, all interior surfaces of the arena were washed thoroughly with 70% ethanol between runs and the cold and warm end walls were alternated every three replicates. To eliminate possible bias from their cooling fans, Both TEC plates were always actively controlled during thermotaxis trials. Robust tick response within the thermotaxis arena required that ticks be actively displaying the host-seeking behavior known as questing, which is stimulated by CO_2_ in the breath of hosts [14, 15]. Questing of tick test subjects was induced by brief agitation of the holding vial and exposure to the breath of the experimenter. All ticks were questing prior to placement into the arena for thermotaxis bioassays.

The arena was illuminated by two 700 lumen LED floodlights with a color temperature of 4000 K (Utilitech Pro 1-Light, 16 Watt Portable Work Light; Utilitech) trained on the center of the arena at angles of 45 degrees, at a horizontal distance of 65cm from either end of the arena. Thermotaxis trials were filmed from above at 1080i resolution with a Canon rebel T6i camera with an 18-55mm zoom lens (Canon, Tokyo, Japan). Each 5-minute-long MP4 video file from a run was transferred to a computer and converted to a stack of 150 JPEG images at 2-second intervals, which were loaded, in chronological order into ImageJ [16]. The position of each tick along the longitudinal axis of the arena was tracked using the MTrack3 plug-in and reported every 2s in cm, with the warm plate located at 0 and the cold plate at -100 cm.

With the exception of temperature sensitivity trials, the temperature of the target was set to 40 °C, the temperature at which, according to the Stefan-Boltzmann law, a surface with an emissivity of 0.94, as measured for the blue anodized TEC plate surface, would radiate energy at the same intensity as human skin, which has an emissivity of 0.98 [12], does at 37 °C.

Previous investigations that failed to find that ticks [7] or hematophagous insects [17] were attracted by radiant heat used a test tube covered with aluminum foil as the non-emitting source and a similar tube with a layer of cellophane over the aluminum foil as the IR-emitting source. We were skeptical that the thin, transparent layer of cellophane would emit sufficient radiant heat, so we tested this method by wrapping a section of a 1l graduated cylinder with aluminum foil and another section of the same cylinder with aluminum foil covered with cellophane, and filled the cylinder with 37 °C water. The thermal imaging camera measured the temperature of the uncovered glass as 37 °C and that of the aluminum foil section as 21 °C, because the foil emits almost no thermal IR itself, but rather reflects that from the room temperature surroundings. The cellophane over aluminum foil section, which was intended in the cited studies to emit radiant heat, appeared to the thermal imaging camera to be at 24.6 °C (S1 Fig) indicating that the cellophane radiated very little heat and mostly transmitted ambient thermal radiation reflected by the underlying aluminum foil. We can conclude that previous studies failed to show that ticks were attracted by radiant heat because the radiant heat stimulus used was much less intense than intended. Another flaw with this method is that if there had been a warm radiating surface in the arena, its radiation would have been reflected by the aluminum foil, confounding the location of the target. It is important that all surfaces in the arena be non-reflecting.

Several measurements and test conditions were used to determine whether the ticks in the thermotaxis arena were locating the warm TEC plate by radiant heat, air convection or heat conduction along the floor. Heating the TEC plates to 40°C increased the surface temperature of the floor less than 1°C and the air temperature less than 0.1°C, within 2.5 cm of the plate, with no observable change in temperature of either the plywood or air beyond that distance, making it unlikely that ticks could detect heat from the plate by conduction or convection beyond its immediate vicinity. To directly test the ability of ticks to detect radiant heat, we conducted trials with the TEC plate surfaces clad with aluminum foil (emissivity = 0.03) adhered with Glue Stic adhesive (Avery Dennison, Glendale, CA, USA). This would eliminate emission of radiant heat without affecting conductive or convective heat transfer to the ticks. We also conducted trials with IR-transparent 10 cm x 10 cm windows of polyvinylidene chloride film (PVDC, Saran^™^ food wrap) on wire frames placed 2.5cm from the TEC plates, to isolate air near the TEC plates from the rest of the arena and thereby eliminate possible convective but not radiant heat transfer to the tick.

To confirm that the Haller’s organ posterior capsule is the IR sense organ, thermotaxis trials were conducted with unfed virgin adult *A*. *americanum* females before and after application of low-melting dental wax (Kerr, Orange, CA, USA) onto the posterior capsules of both front legs using a hot 0.25 mm diameter silver wire attached to the tip of a soldering iron (Weller, Apex, NC). Care was taken to exclusively place the wax onto the posterior capsule; any wax accidentally applied to the anterior pit sensilla was removed using sterile forceps and a minute piece of kimwipe (Kimberly-Clark, Irving, TX). Following wax application, ticks were allowed to recover for 10 min prior to being tested in thermotaxis trials. We also attempted to localize the IR sense to the forelegs and Haller’s organ by means of amputation, but the questing behavior of the test subjects was greatly reduced.

### Repellent thermotaxis bioassays

The effects of repellents on thermotaxis was assessed by introduction of known concentrations of repellent vapors into the closed air circulation system of the arena. An in-line low-pressure air circulation fan (Intex, New Delhi, India) pulled air from the arena through 1.5 cm-diameter screened exhaust ports, located midway up the side walls, 10 cm from each TEC plate, and re-introduced a combined airstream into the center of the arena through a screened 1.5 cm-diameter inlet port, at a rate of 900 ml/min (Fig 1). Reagent grade repellents N, N-diethyl-meta-toluamide (DEET), 2-undecanone, picaridin, citronellal and nootkatone (Sigma Aldrich, St. Louis, MO, USA) were purchased 2 weeks prior to use and stored at room temperature. Repellents were diluted into 95% ethanol on the day of use so that 0.5 μl would give the desired concentration in the 10 l arena, which was applied to the tip of a temperature-controlled soldering iron and vaporized directly into the circulating air through an entry port in the suction tubing (Fig 1) by briefly turning the tip temperature up to 65°C. Control repellent thermotaxis trials were performed with 0.5 μl 95% ethanol. Ticks were pre-exposed to the same control and repellent vapor concentration for 2 min in a 1l glass bottle prior to placement into the bioassay arena. Control and repellent thermotaxis trials used three runs of eight unfed virgin adult *A*. *americanum* females, unless otherwise noted.

### Y-tube olfactometer bioassays

Y-tube olfactometer bioassays were conducted as previously described [15] to determine whether concentrations of repellents that disrupt thermotaxis also affect tick olfaction, another function ascribed to Haller’s organ. Tests were conducted during the day between the hours of 11:00–15:00, at 23 ± 1°C and 40% R. H., under ambient (LED) lighting. Each arm of the Y-tube olfactometer (S2 Fig) was supplied with either breathing quality air or 3% carbon dioxide (CO_2_) in breathing quality air (AirGas, NC, USA), at a flow rate regulated and measured with a #11 Compact Shielded flow meter (Gilmont Instruments, Barrington, IL, USA). A vacuum pump removed gases from the downwind end of the Y-tube at a rate equal to the total flow through the olfactometer and exhausted them out of the test area. Ticks were pre-exposed to repellent vapors for up to 5 min, as previously described, prior to placement into the Y-tube, with no additional repellent exposure during Y-tube bioassays. 50ng/cm^3^ and 100ng/cm^3^ DEET, which are 1X and 2X, respectively, the concentration found to completely abolish thermotaxis, were evaluated in Y-tube bioassays. After repellent pre-exposure, ticks were immediately placed at the Y-tube starting mark and their movements were filmed for 5 min. Olfactometer bioassays consisted of six runs of eight unfed virgin adult *A*. *americanum* females randomly selected from a total population of 250 and acclimated to experimental conditions for 20 min, prior to repellent pre-exposure. To eliminate contamination and positional bias, all Y-tube components were washed thoroughly with 70% ethanol between replicates and the CO_2_ gas was alternated between the two arms of the Y-tube. Ticks were recorded as positive responders if they made a choice in Y-tube bioassays, moving 1 cm past the choice point (S2 Fig). Trials were filmed and scored as previously described and those data were analyzed using the Generalized Linear Model in R, as described below.

### Epi-Microscopy

The Haller’s organ posterior capsule was imaged with a Keyence VHX-5000 digital microscope using epi-illumination with polarized light and a VH-Z500R 500X-5000X objective (Keyence Corporation, Elmwood Park, NJ, USA). A Woodpecker UDS-J ultrasonic dental scaler (Guilin Woodpecker Medical Instrument Co., Guilin, China) was modified for micro-etching of the cuticle to remove the cover from the Haller’s organ posterior capsule for imaging. The stainless-steel tip supplied by the manufacturer was sharpened mechanically with a hard Arkansas whetstone wet with mineral oil, and then further electropointed in a solution containing 34 ml 98% sulfuric acid, 42 ml 25% orthphosphoric acid and 24 ml deionized water. A 6 VDC source was used with the variable resistance adjusted to control the rate of etching so that a sharp, polished tip could be obtained [18]. It was furthermore necessary to reduce the power supplied to the ultrasonic generator handpiece by placing a 3 KΩ, 5W resistor in series with it. The handpiece with sharpened tip was fixed to a micromanipulator to perform manually controlled etching of the cuticle covering of the Haller’s organ posterior capsule.

### Statistical analysis

For scoring a thermotaxis assay as a choice test, ticks located within 10 cm of the warm plate (red area in Fig 1) at the end of the 5 min trial were counted as warm plate responders, while those ending up within 10 cm of the cold plate (blue area in Fig 1) were counted as cold plate responders. Ticks that did not reach either the warm or cold regions were counted as “neither” or “no choice”. Statistical analysis of choice data for each trial was performed using the generalized linear model [19] with binomial distribution and logit link function. Data were subjected to generalized linear mixed model ANOVA based on the model logit(Y_ij_) = U + T_i_ + e_ij_, using function glm in R3.3.3 (R Core Team 2017). In this model, Y_ij_ (Y = 0,1) is the index of tick j (j = 1,2,3… n) choice i (i = 1,2,3), U is the average preference (probability) of tick j, T_i_ is the choice (preference) i effect, and e_ij_ is the residual error.

Estimated choice preference means were back-transformed to proportions. Pair-wise comparison results were given as response scale (i.e. difference of proportions, not log odds ratio). Significance of the overall preference effect was evaluated using a Chi-square test of deviance, and comparison of two choice preference proportion in terms of log odds ratio was conducted using a Wald test with z-values. P value adjustment: Tukey method for comparing a family of 3 estimates. A significance level of α = 0.05 (confidence level 95%) or better was used for all statistical tests.

## Results

### Ticks are attracted to a warm surface from a distance

**Fig 2:**
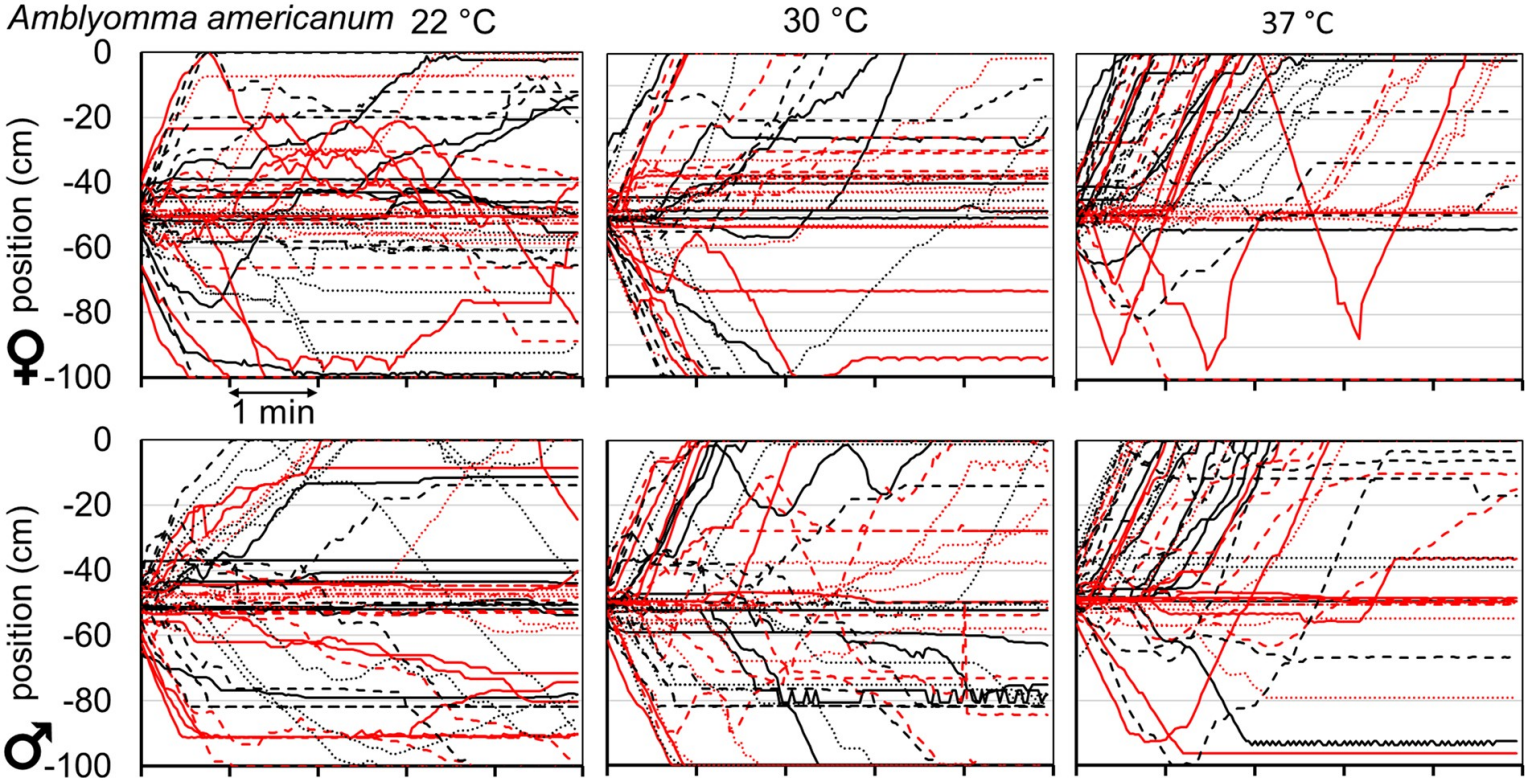
Tracks of individual *Amblyomma americanum* ticks in thermotaxis trials. Longitudinal position vs. time in the 1m-long arena of 48 individual female (top) or male (bottom) *A*. *americanum* ticks (six runs of eight ticks, with a different color/line type combination for each run) after placement in the center, at -50 cm, with the target plate (22, 30 and 37 °C in left, center and right panels, respectively) at 0 cm and the cold plate at -100 cm. Horizontal axis units are minutes.

**Fig 3:**
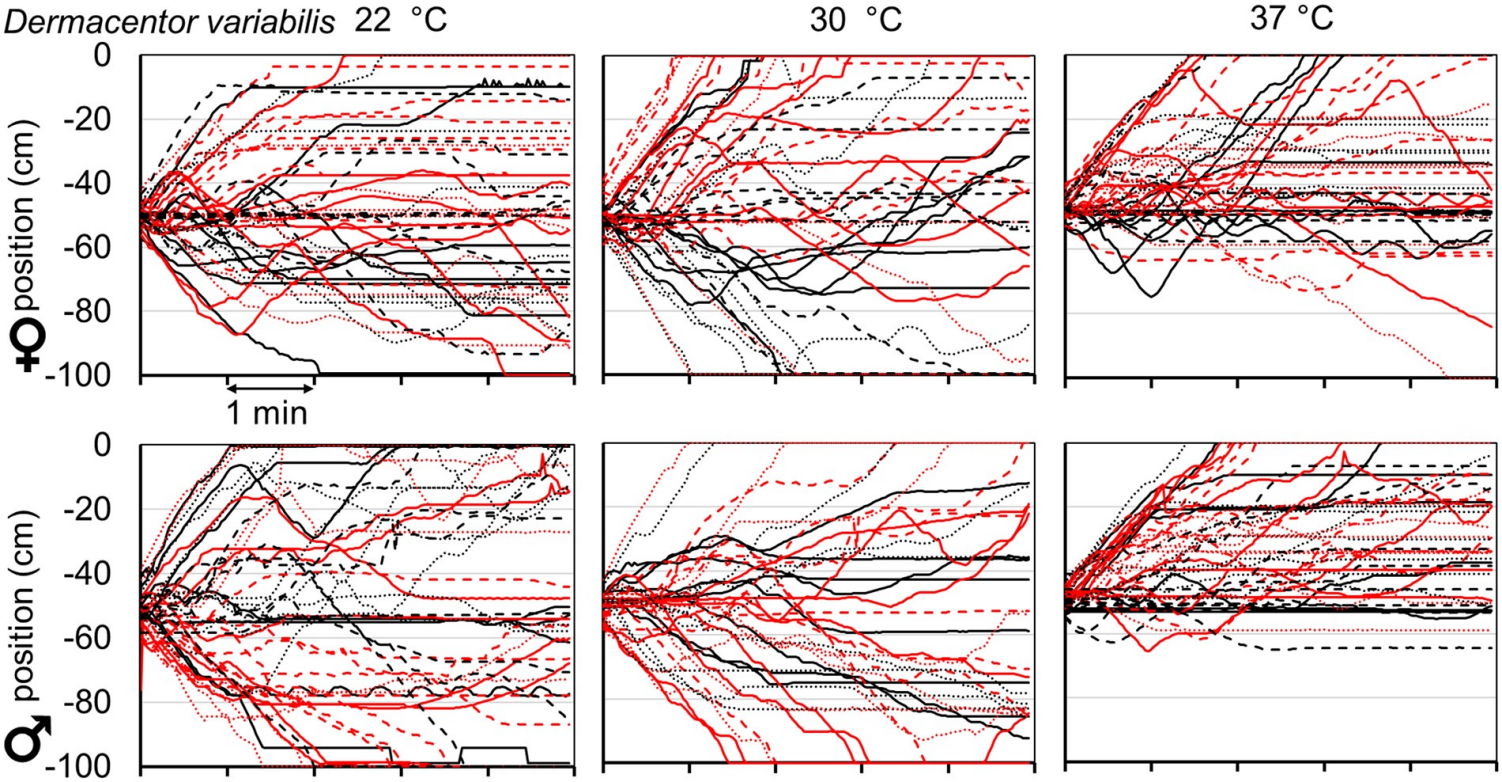
Tracks of individual *Dermacentor variabilis* ticks in thermotaxis trials. Longitudinal position vs. time as in Fig 2, but for *D*. *variabilis* ticks.

**Fig 4:**
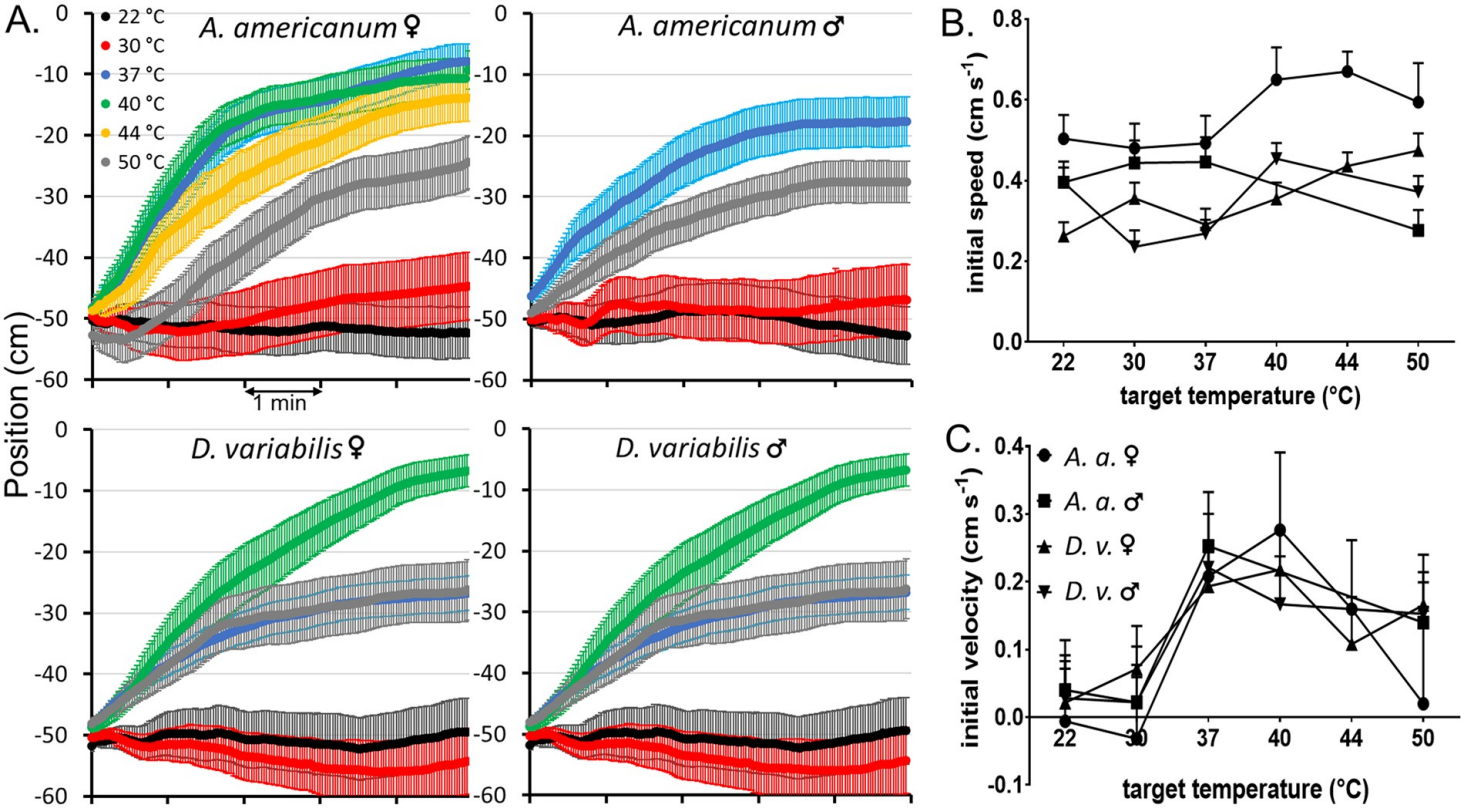
Average movement of ticks in thermotaxis trials. A. Average longitudinal position vs. time in the thermotaxis arena of the indicated ticks after placement in the center at -50 cm with respect to the warm target at 0 cm. Horizontal axis units are minutes. B. Average initial speed and C. average velocity of ticks during the first 10 seconds of thermotaxis trials.

**Fig 5:**
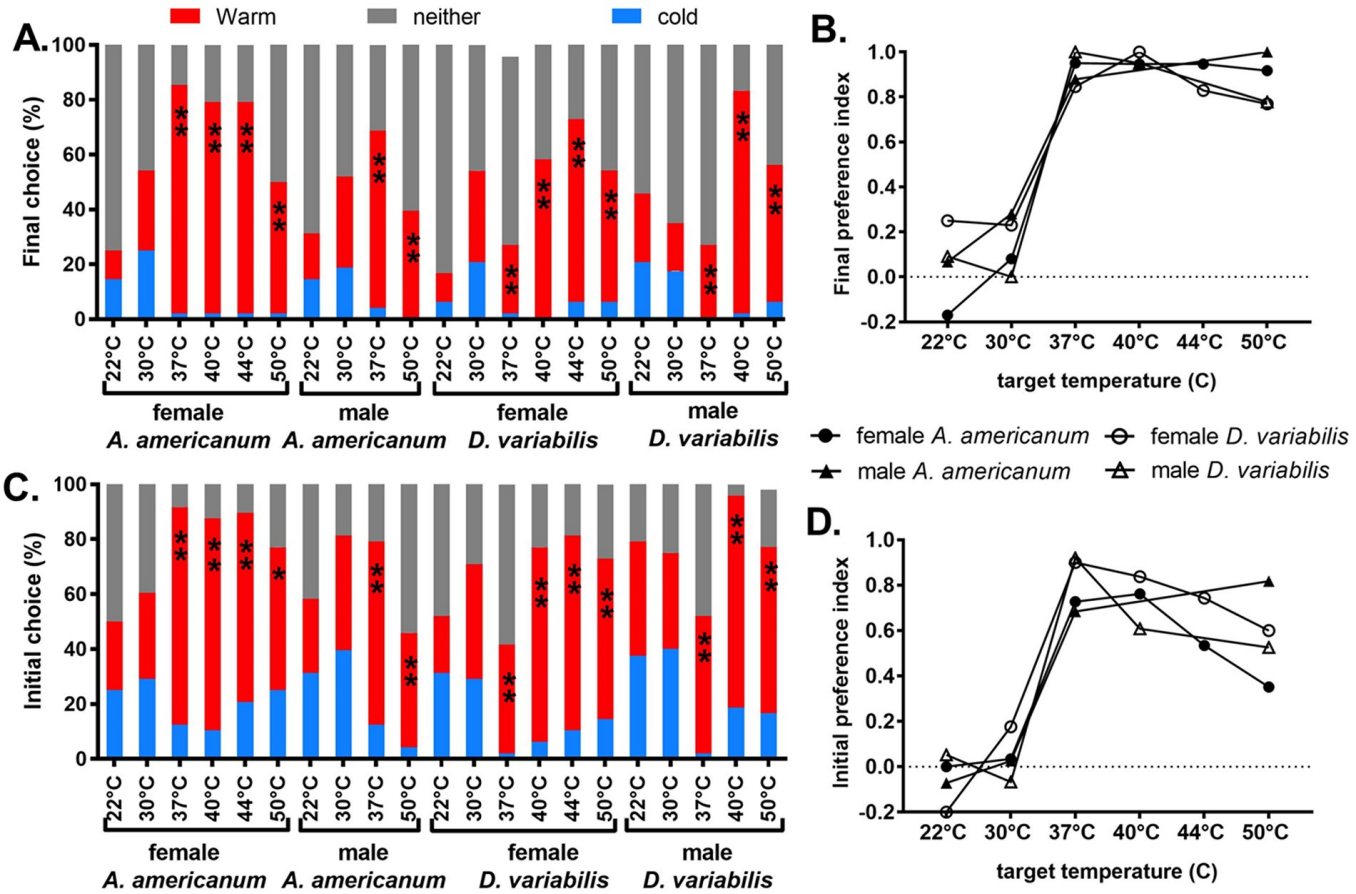
Thermotaxis trials scored as choice tests. (A) Final choice of ticks at the end of a 5 min trial was scored as “warm” or “cold” if within 10 cm of the corresponding end, or “neither” if between those end areas. (B) Final warm preference index (PI), defined as (warm-cold)/(warm+cold) [20]. (C) Initial choice scores based on direction of the first movement of more than 15 cm if the tick eventually moved at least 25 cm from the origin. Ticks that failed to move at least 25 cm were scored “neither”. (D) Initial warm PI. Significance of warm/cold difference: **: p < 0.001; *: p < 0.05.

Questing unfed virgin adult *A*. *americanum* and *D*. *variabilis* ticks of both sexes were strongly attracted to an anodized aluminum plate warmed to a temperature of 37 °C or higher (Figs 2–4). For example, when questing unfed virgin adult *A*. *americanum* female ticks were placed in the center of the arena, an approximately equal number moved in each direction and many ticks did not move toward either plate, when both plates were at a temperature of 22 °C (Fig 2, upper left and S1 Movie). When the warm plate was at 30 °C (Fig 2, upper middle), the results were similar. Most of the ticks that initially moved toward the cold plate or the warm plate kept moving in the same direction and only slightly more ticks ended up within 10 cm of the warm plate at the end of the 5-minute trial, but the differences were not significant (Fig 5A). When the warm plate was at 37 °C, on the other hand (Fig 2, upper right and S1 Movie), most ticks began moving toward it almost immediately, and of the few that did initially move toward the cold plate, most reversed direction and headed toward the warm plate, so that after five minutes, only one of 48 female *A*. *americanum* ticks remained within 10 cm of the cold plate, while 40 were within 10 cm of the warm plate. Seven ticks were between these end regions and were counted as not having chosen either the warm or the cold plate. Results were similar for male *A*. *americanum* and also for both sexes of *D*. *variabilis* (Figs 2 and 3).

The average position as a function of time in thermotaxis trials for both sexes of both tick species at all temperatures tested is shown in Fig 4A. The ticks began moving toward the warm plate immediately when it was at 37, 40 or 44 °C, but when it was at 50 °C, average movement toward the warm plate was decreased and delayed. While average initial velocity in the direction of the warm plate during the first 10 seconds increased from essentially zero at 22 and 30 °C to maximal at 37 and 40 °C, indicating that ticks could easily detect the warm plate from a distance of 50 cm (Fig 4C), average initial speed did not vary consistently with temperature (Fig 4B), indicating that temperature does not stimulate movement, but does determine the preferred direction of movement. At warm plate temperatures of 44 and 50 °C, average initial velocity and average net movement toward it were less than maximal (Fig 4A and 4C).

We analyzed the tick thermotaxis trials as choice tests, scoring the choice as “warm” or “cold” if the final position of the tick at the end of the five-minute trial was within 10 cm of one of those plates and “neither” if it was between those areas (Figs 1 and 5). Although the fraction of ticks choosing neither side might be expected to decrease with temperature, this was not a consistent response, and the observed variability in this parameter might be due to uncontrolled factors, such as variation in responsiveness during the day or between days. Despite the variability in the fraction of ticks making a choice, there was a clear and consistent temperature dependence of preference for the warm plate when its temperature was 37 °C or higher. While there was no significant difference in the fraction of ticks choosing the warm vs. the cold plate when the “warm” plate was at 22 or 30 °C, this difference was highly significant (p< 0.001) at all higher temperatures tested, for both sexes of both species (Fig 5A).

An index of warm plate preference was defined as (warm-cold)/(warm+cold) and could vary between -1 and +1, with 0 indicating no preference [20]. The final choice preference index was near zero for both sexes of both tick species at 22 and 30 °C, increasing to near 1 at temperatures of 37 °C and higher. It remained at or near 1 for both sexes of *A*. *americanum*. ticks up to 50 °C but declined slightly for both sexes of *D*. *variabilis* ticks at 50 °C (Fig 5B).

To analyze the initial preference of ticks for the warm plate, we defined an initial movement of more than 15 cm in the direction of either plate as an initial choice for that plate if that tick eventually moved at least 25 cm from the origin during the 5-minute-long trial. Ticks that failed to move at least 25 cm were counted as having chosen neither plate. Initial preference (Fig 5C) showed overall trends similar to those of final preference. There was no significant difference in initial preference for the warm vs. the cold plate at 22 or 30 °C, whereas the preference for the warm plate was highly significant at all higher temperatures for both sexes of both tick species. The fraction of ticks scored as having made a choice was higher for initial preference than for final preference in all trials, because of the lower threshold for a choice of 25 cm compared to 40 cm from the origin; some ticks stopped between these lines before the end of the 5-minute trials.

As with the final warm/cold preference index, the initial preference index was near zero for both sexes of both tick species at 22 and 30 °C and increased greatly at 37 °C and above (Fig 5D). Compared to final preference, initial preference showed a sharper optimum, between 37 and 40 °C, with a greater decrease at the higher temperatures for three of the groups but not for *A*. *americanum* males, which showed a higher preference index at 50 °C than at 37 °C. The decreased thermotaxis at temperatures above optimum reflects real preferences of the ticks and not confusion caused by possible reflections of radiation from the warm plate in the cold plate, as such reflections were undetectable with the thermal imaging camera at the highest temperatures used. The sharp temperature dependence of the initial preference index indicates that at least 70 to 90% of males and females of both tick species can detect the warm plate from a distance of 50 cm.

Because males and females of both tick species showed strong thermotaxic behavior, we focused on only a single type, female *A*. *americanum*, for subsequent experiments. Furthermore, we set the temperature of the warm plate to 40 °C, at which, according to the Stefan-Boltzmann law, a surface with an emissivity of 0.94, as we measured for the blue anodized TEC plate surface (see Methods), would radiate energy at the same rate per unit area as human skin at 37 °C, which has an emissivity of 0.98 [12].

### Distance sensitivity of temperature detection

**Fig 6:**
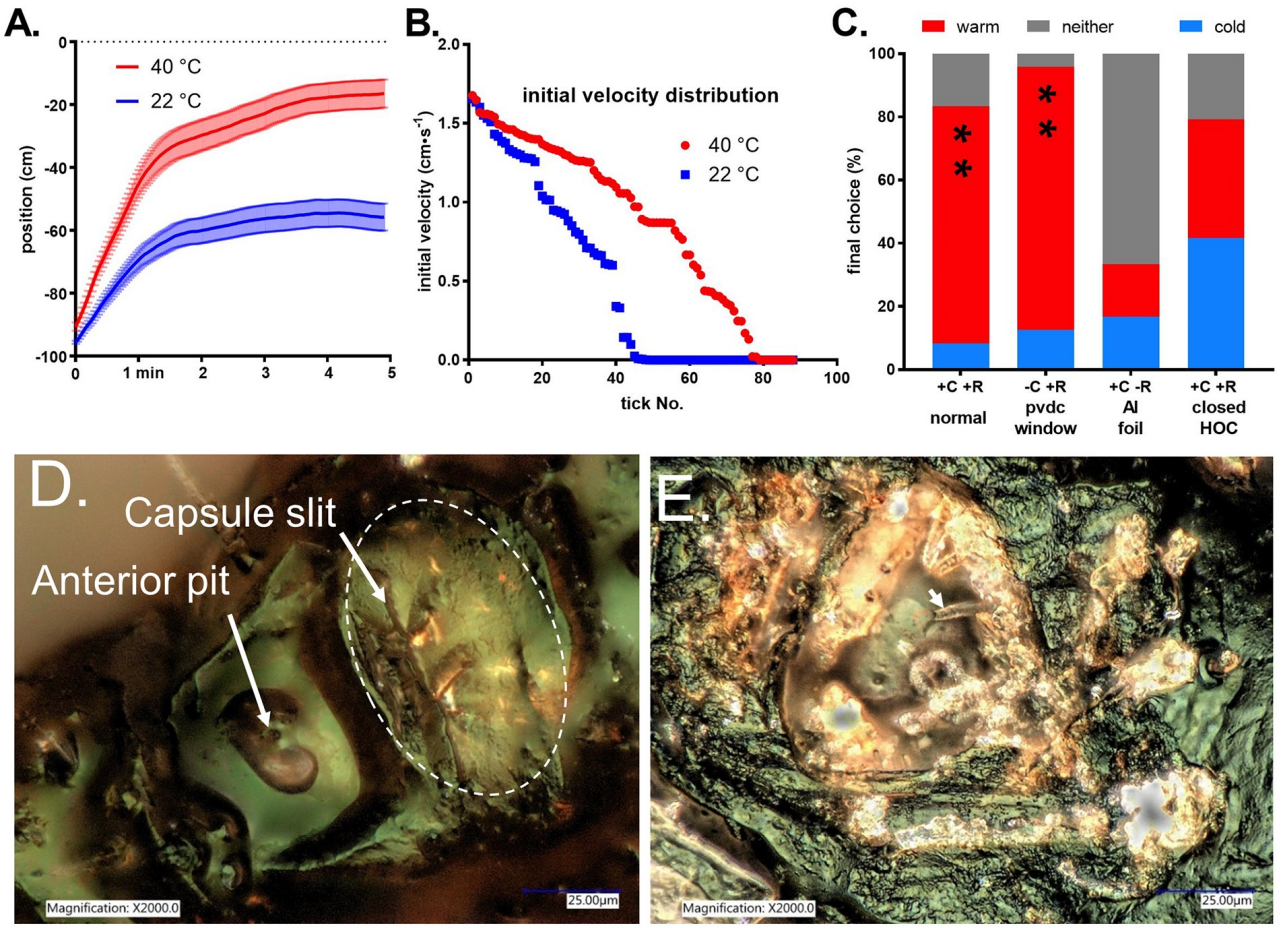
Sensitivity and mechanism of tick heat sensing. A. Mean longitudinal position (± SEM) over time (s) of 88 *A*. *americanum* females (measured in 11 groups of 8) after placement adjacent to the cold end wall at -100 cm with respect to the target wall, which was set to either 22 or 40 °C. B. Distribution of initial velocities of all 88 ticks tested at 22 or 40 °C, averaged over the first 10 s, from fastest to slowest. C. Final choice scores in thermotaxis trials of *A*. *americanum* females placed in the center of the arena, with the target at 40 °C, under normal conditions, where convection and radiation from target to ticks are both possible (+C +R, normal), with an IR-transparent PVDC film barrier 5 cm from each end wall to block any possible convective air currents (-C +R, PVDC window), with both end walls clad with aluminum foil to eliminate radiant heat emission and leave only the possibility of heat transfer by convection (+C -R, Al foil), and finally in the normal arena but with both Haller’s organ capsule apertures closed with paraffin wax (+C +R, closed HOC). (** = p < 0.001 for warm/cold difference). D. Female *A*. *americanum* Haller’s organ viewed with epi-illumination shows hints of reflected light through the capsule aperture. Approximate outline of the capsule is shown by dashed oval. E. Interior surfaces of the Haller’s organ capsule and cover debris appear highly reflective after cover removal with an ultrasonic micro-etcher, but thin-walled sensilla (white arrowhead) are not reflective.

The maximum distance at which ticks could detect the warm plate was of interest, but 50 cm was clearly too close to assess this, so we started *A*. *americanum* females at the cold plate, 100 cm from the warm plate. With both plates at room temperature, many ticks began walking immediately toward the far plate, ending up, on average, near the center of the arena, at an average position of -55 ± 4 (mean ± SEM) cm. Most of the distance was covered within the first two minutes (Fig 6A). When the temperature of the far plate was increased to 40 °C, the average movement of the ticks toward it was much faster from the start, indicating a clear preference, even at the distance of 1m (Fig 6A). The ticks reached an average distance of only 16.5 ± 4.5 (mean ± SEM) cm from the warm plate during the five-minute trial. Clearly, many of the ticks could detect the 10 cm X 10 cm warm plate from 1 m away and began thermotaxing toward it immediately.

The initial velocities of all 88 ticks tested at each temperature, averaged over the first 10 s, were sorted and plotted from largest to smallest in Fig 6B. When the far plate was at room temperature, 45 of the ticks showed some initial movement toward it. These could be considered to be moving spontaneously. When the warm plate was at 40 °C, 76 of the 88 ticks began moving towards it immediately. Of these, we can assume that 45 were moving spontaneously and the remainder, 31, were thermotaxing. Since 31 of the 43 ticks that were not moving spontaneously, or 72%, began thermotaxing immediately from 1m away, we can conclude that at least 72% of adult *A*. *americanum* females can detect the 10X10 cm plate at a distance of 1m, just as many as a t 0.5 m. A longer arena or smaller target is needed to precisely assess the range of tick heat detection, but we can conclude that since an adult human torso is approximately 4 times the width of our target, a tick could detect an adult human at a distance of up to 4 meters under ideal conditions.

### Mode of tick thermosensation

From the results presented so far it is clear that *A*. *americanum* and *D*. *variabilis* ticks can detect heat at distances that could only reasonably be achieved by IR-sensing, but it was important to firmly establish the role of IR-sensing and to eliminate the possibility of thermosensation by conduction or convection. Conduction of heat along the plywood floor and sides of the arena was eliminated by temperature measurements (see Materials and methods). Furthermore, elimination of the possibility of convective heat transfer from the plate to the ticks by placing an IR-transparent plastic film barrier had no significant impact on the percentage of ticks choosing the warm plate (Fig 6C). However, when the plates were clad with aluminum foil with very low emissivity, which effectively eliminated emission of thermal radiation without affecting conduction or convection, the ticks no longer showed a preference for the warm plate (Fig 6C). These results confirm that the ticks sense heat at a distance by IR-sensing.

As discussed in the introduction, the anatomy of the Haller’s organ capsule suggested that it could be a directional IR sensor. That the Haller’s organ capsule was essential for thermotaxis was confirmed by the finding that *A*. *americanum* females with their Haller’s organ capsule apertures occluded with paraffin wax showed no preference for the warm plate in thermotaxis trials (Fig 6C).

In order for the sensilla in the Haller’s organ capsule to selectively sense the temperature of an outside source via thermal radiation entering through the capsule aperture and not the tick’s own body temperature via thermal radiation from the capsule walls, the capsule walls and cover must not emit IR but instead must reflect incident IR onto the sensilla. When we removed the capsule cover with an ultrasonic etcher (see Materials and methods), we noticed that the interior of the capsule appeared bright and reflective in a binocular microscope. Viewing female *A*. *americanum* Haller’s organ capsules with epi-illumination, the aperture of the intact capsule appears to emit bright points of reflected light and reflected light also appeared from several other points on the capsule cover, presumably where the cuticle was thinner (Fig 6D). When the cover of the capsule was removed with the ultrasonic microetcher, much of the inside of capsule and the inside faces of cover fragments scattered on the surrounding cuticle appeared highly reflective (Fig 6E). The reflective areas inside the rim of the open capsule in Fig 6E appear to be the pleomorphs previously described in ultrastructural studies [9, 10]. One thin-walled sensillum or conical seta is shown by the white arrow and is not reflective. Other setiform structures in the image are visible but appear reflective and may be cylindrical setae described by Bruce [9, 10].

### Repellents abolish tick thermotaxis

**Fig 7:**
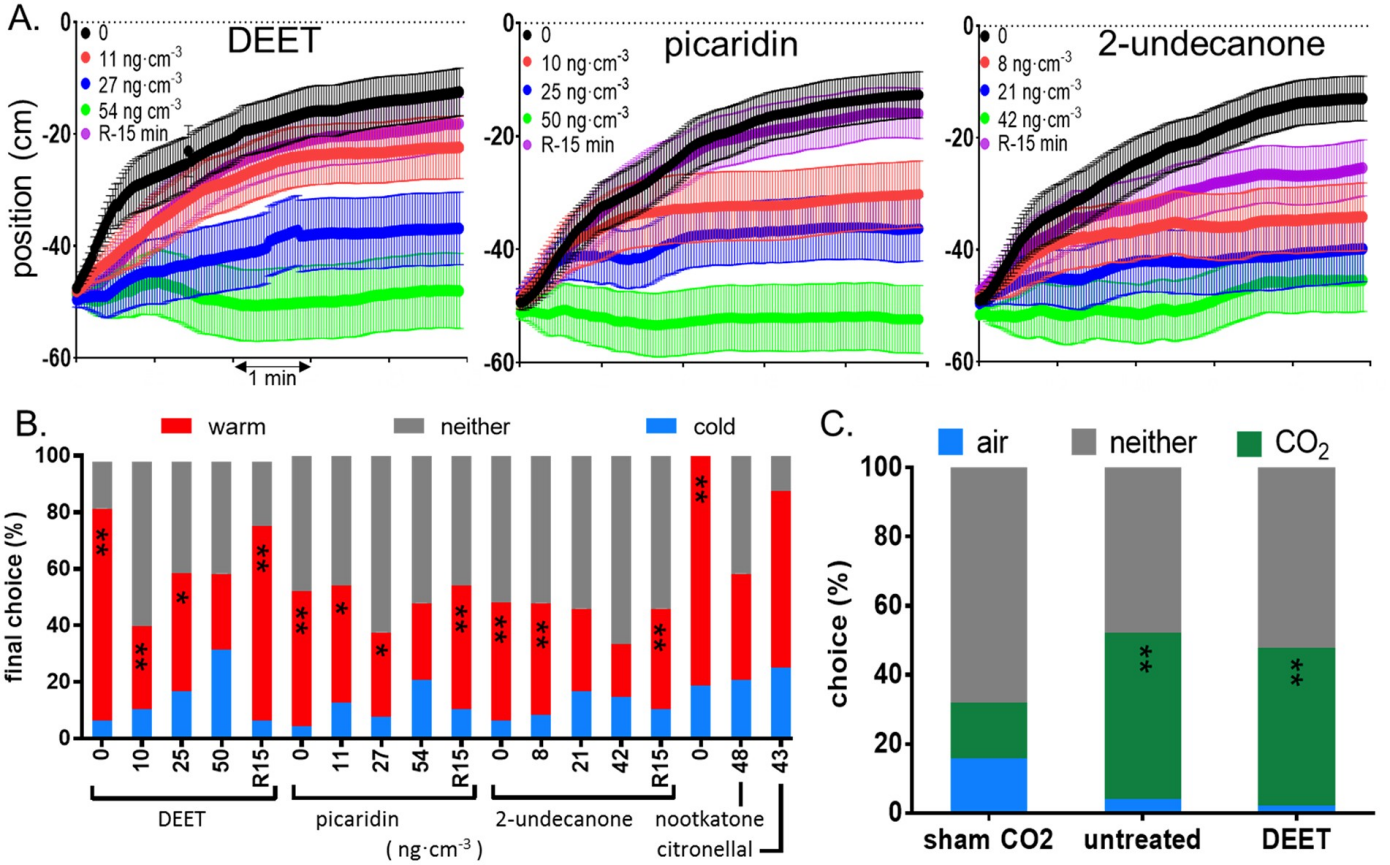
Disruption of thermotaxis by repellents. (A) Mean longitudinal position (± SEM) in thermotaxis trials of unfed virgin adult *A*. *americanum* females exposed to the indicated repellent vapor concentrations (ng•cm^-3^) or after 15 minutes’ recovery from the highest concentration (R). Ticks were placed into the center of the arena, at -50 cm with respect to the 40 °C target wall. (B) Final position after 5 minutes, scored as in Fig 5A. (C) Y-tube choice assays with unfed virgin adult *A*. *americanum* females under three conditions. Sham CO_2_: both tubes contained pure air. Untreated: air in one tube contained 4% CO_2_. DEET: air in one tube contained 4% CO_2_ and all ticks were exposed to 50 ng•cm^-3^ DEET. Significance of warm/cold or CO_2_/air difference: **: p<0.001; *: p<0.05.

Because of the clear importance of thermotaxis in host-seeking, we ran thermotaxis trials with unfed virgin adult female *A*. *americanum* ticks in air containing DEET, picaridin, 2-undecanone, citronellal and nootkatone, to determine whether these commercial repellents disrupted thermotaxis. Scoring final position after 5 minutes as a choice assay, 50 ng•cm^-3^ DEET, 54 ng•cm^-3^ picaridin and 42 ng•cm^-3^ 2-undecanone completely abolished thermotaxis, and the effect was completely reversible within 15 minutes (Fig 7, S3 Fig and S1 Movie). Thermotaxis was also disrupted by citronellal and nootkatone, but because of the difficulty in removing the odor of these compounds from the arena, only the highest concentration of each compound was tested, with only eight and 24 ticks, respectively (Fig 7B).

Motion of individual ticks exposed to the highest concentrations of DEET, picaridin and 2-undecanone in thermotaxis trials with the warm surface at 40 °C was similar to the trials in Figs 2 and 3 with the warm plate at 22 °C (S2 Fig). In these cases, most of the ticks began moving as soon as they were placed in the arena and showed a tendency to continue moving in the direction in which they started, but there was no clear preference for either plate after repellent treatment. Time courses of average position showed that the repellents dose-dependently reduced the net movement toward the warm plate (Fig 7A); At the highest concentration of all three repellents tested, there was no net movement toward either plate. Final preference index and fraction of ticks making a choice for either plate within five minutes for the repellent trials are plotted in S3B Fig. The concentrations that reduced preference index to 50% of control were estimated as 26, 31 and 17 ng•cm^-3^ for DEET, picaridin and 2-undecanone, respectively. While there was a slight trend toward fewer ticks making a choice with increasing concentration of DEET exposure, this was not the case with picaridin or 2-undecanone. Initial speed during the first 10 seconds of the trials did not vary consistently with repellent concentration (S3C Fig), indicating that repellents do not affect the number of ticks that are host-seeking, but only the direction of movement.

Because host-seeking behavior is stimulated by olfactory cues like CO2, we directly examined the effect of DEET on the response to CO_2_ in a Y-tube. When starved virgin adult female *A*. *americanum* ticks were placed in the Y-tube with air flowing in both tubes, 32% of ticks made a choice, with approximately equal numbers going to either side (Fig 7C, Sham CO_2_). When the air in one tube contained 4% CO_2_, 52% of ticks made a choice, with most choosing the side with CO_2_. Exposure to 50 ng•cm^-3^ DEET before and during the trial did not affect the ability of the ticks to choose the airstream containing CO_2_ (Fig 7C).

## Discussion

While it has been known that ticks and other ectoparasites are attracted to heat, previous workers concluded that ticks [7], mosquitoes[21] and lice [17] sensed warm air currents and not radiant heat. However, we have shown in the materials and methods section of this paper that the single published previous attempt to assess attraction of ticks to radiant heat [7] could not have worked because the strategy of wrapping a non-emitting aluminum foil surface in cellophane to make it emitting did not make it a strong enough emitter. An even earlier study found that the triatomine bug *Rhodnius prolixus* was attracted to heat, but the fact that no preference was found for a test tube coated with lamp black over a clean one led to the conclusion that warmth was sensed by convection and not radiant heat. However, this experiment was also flawed, based on the mistaken premise that “the radiant heat from the blackened tube is many times greater than that from the clean tube”[22], whereas in fact the emissivity of pyrex glass, 0.97, is greater than that of lamp black (0.95)[23]. More recent work has shown that triatomine bugs are indeed attracted to radiant heat [24, 25].

When we used a warm surface with emissivity close to that of human skin, we found that both sexes of two ixodid tick species, the lone star tick *A*. *americanum* and the dog tick *D*. *variabilis*, have exquisitely sensitive thermal IR detectors that enable them to thermotax toward a warm object with a temperature optimum in the range of the body temperatures of their warm-blooded mammalian and avian hosts, and have estimated that female *A*. *americanum ticks* have the sensitivity and directionality to detect the thermal IR from a human body at a distance of up to 4 meters.

We used heat from a warm anodized aluminum plate rather than an electronic IR source as the stimulus for thermotaxis. This is preferred, since the warm surface closely mimics the spectrum and intensity of IR that would be emitted by human skin at the same temperature, allowing the determination of the temperature sensitivity and range of the IR sense. It would be unnecessarily complicated to try to mimic this radiation spectrum with a non-thermal IR source.

We have shown that female *A*. *americanum* ticks detect and navigate toward a 10 X 10 cm warm planar source from a distance of at least 1 m, using thermal IR sensing. Simply scaling up to the size of a human torso, which would be at least four times as wide as the 10-cm plate, allows us to say that under ideal conditions, ticks could detect an adult human at a distance of at least 4 meters. When stimulated to quest, ticks seemed to walk at a constant speed that did not vary consistently with temperature, indicating that temperature does not stimulate movement, but determines the preferred direction of movement. The fastest walking speed of female *A*. *americanum* ticks recorded in the experiment of Fig 6B was 1.65 cm•s^-1^, which is equivalent to the 1 m•min^-1^ speed observed for *Hyalomma* ticks hunting in the field [4]. The fastest walking speeds we observed for *A*. *americanum* males, *and D*. *variabilis* females and males were 1.5, 1.2 and 1.1 cm•s^-1^, respectively.

This newly discovered thermal-IR sense is located in the Haller’s organ capsule, a camera-lucida-like sensory organ on the 1st walking leg. Historically, Haller’s organ, including its capsule, was thought to be primarily olfactory [4, 6–8]. While we were preparing this manuscript, a paper was published showing that the tick Haller’s organ is required for positive phototaxis toward near-infrared light with a wavelength of 880 nm, and it was postulated that the capsule is an IR sensor [11]. While 880 nm is well outside the range of thermal IR emitted by human skin, which includes wavelengths between 5 and 20 μm, with a peak near 10 μm [12], it is likely that the tick thermal IR sensors were warmed enough by this light to stimulate thermotaxis. However, we demonstrate here for the first time that the capsule is a thermal IR sensor and that it has an overriding importance in host-seeking thermotaxic behavior.

Since thermal IR is well outside the spectral sensitivity of photoreceptors, we propose that the sensilla in the capsule are thermosensors that are warmed by thermal radiation. The capsule of Haller’s organ seems optimally designed to be a directional IR sensor, with the aperture restricting the direction from which radiation is perceived. Since adult *A*. *americanum* females can reliably detect the 10X10 cm plate at a distance of 1m by radiant heat, the field of view of their heat sensor is at most 5.7 degrees. We have also shown for the first time that the interior surfaces of the capsule are highly reflective to visible light and presumably also to IR, which is essential for high sensitivity to incident radiation without interference from radiation emanating from the surrounding capsule surfaces. Further work is needed to measure the spectral properties of this reflectivity and to investigate the reflective mechanism of this special cuticle.

**Fig 8:**
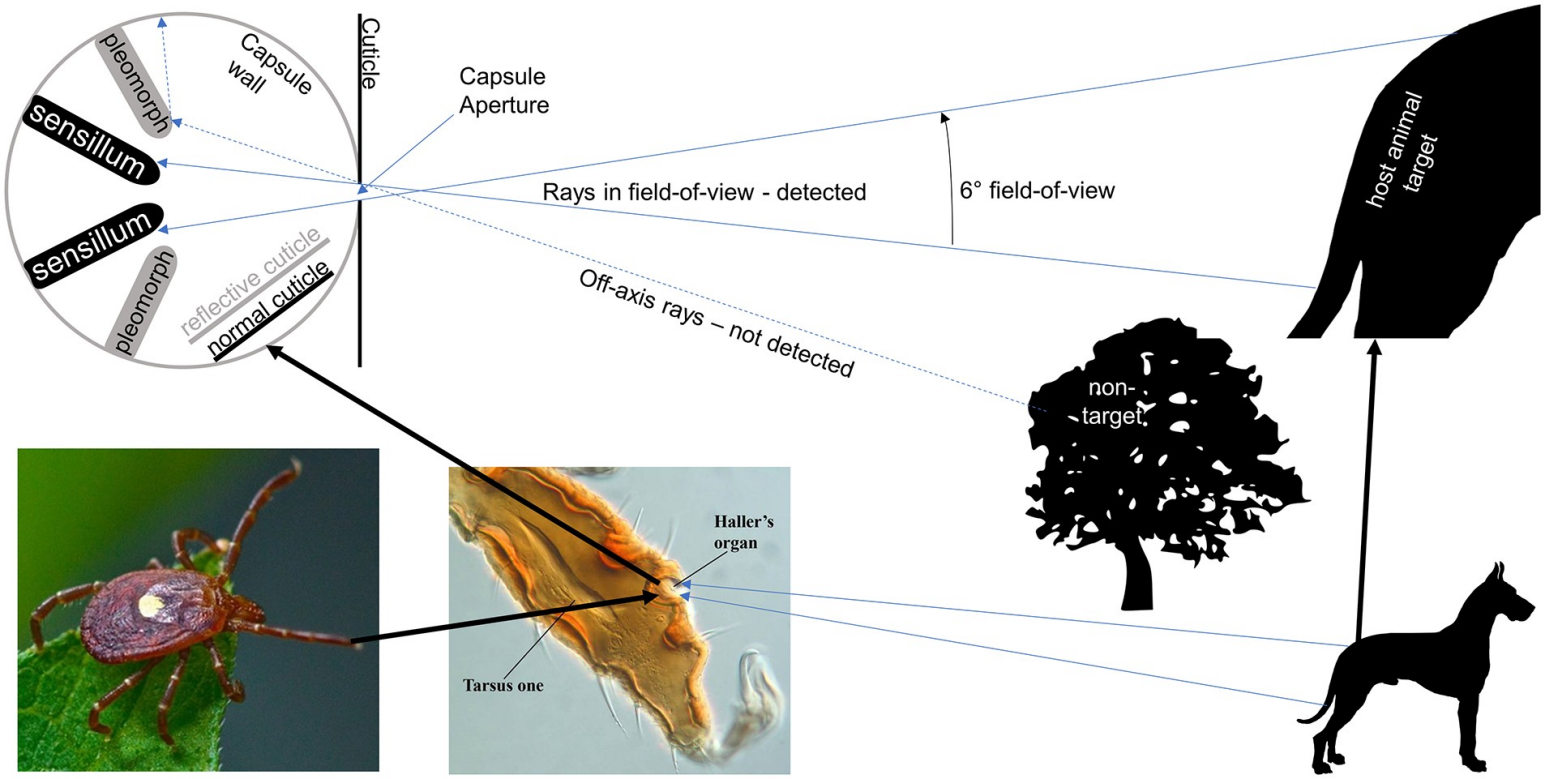
Proposed function of the Haller’s organ capsule as a radiant heat sensor. When the aperture of the the Haller’s organ capsule near the tip of the foretarsus is directed at a warm target, infrared radiation within a narrow field of view passes through the aperture and warms the sensilla located deep within the capsule, whereas off-axis rays from non-target areas will be blocked by reflective pleomorphs and not influence the sensilla. The sensilla will be warmed to the average temperature within the field of view. The walls of the capsule and the pleomorphs are reflective and so will not radiate to the sensilla but instead will insulate them. Reflective cuticle is grey and normal cuticle is black. Image of female lone star tick, *Amblyomma americanum*, on leaf from Ticksafety.com, with permission. Differential interference contrast micrograph of the foretarsus of a larval *Haemophysalis* tick, from [26], with permission from W. Knee and H. Proctor.

We propose that the Haller’s organ capsule functions as a highly directional radiant heat sensor as shown in Fig 8. Two of the eight previously described [9, 10] thermosensitive sensilla are shown deep in the capsule and these are shielded by more superficial pleomorphs, which, as seen in light and scanning-electron micrographs, seem to form a circle above the sensilla [9, 10]. The function of these odd-shaped structures was previously unknown, but as seen in Fig 6E, they are highly reflective and we propose that they serve to shape the thermal field-of-view of the organ by excluding entry of off-axis infrared rays into the inner thermosensory region of the capsule. The precise shapes of the pleomorphs and the aperture might also have physical significance, but that is beyond the scope of this paper. It is also important that the interior surfaces of the capsule are IR-reflective so that their thermal emission to thermosensilla is minimized.

We have also shown that thermal-IR-guided thermotaxis behavior of ticks is disrupted by the repellents DEET, picaridin, 2-undecanone, citronellal and nootkatone at concentrations of 20 to 50 ng•cm^-3^. Disruption of tick thermotaxic host-seeking behavior by repellents was reversible, and recovery was nearly complete within 15 minutes. To put these concentrations in perspective, 50 ng•cm^-3^ ng DEET is equivalent to 0.5 μl in the entire 10 l arena. It is difficult to compare these concentrations with other repellent data, since most tests evaluate oriented movement away from the repellent [27] and we were interested in the disruption of thermotaxis behavior by repellent exposure. Nevertheless, the comparatively much larger amounts of repellents used in such contact bioassays, usually several hundred μl applied to a filter paper or other surface [28], would certainly result in volatilization of enough material to disrupt thermotaxis of ticks that approach the material, and this might appear to be repellency. On the other hand, such large amounts of material may indeed be repellent by a thermosensory-independent mechanism, such as irritancy or disruption of olfaction.

The behavior of ticks after release in the arena with a warm plate at one end, after exposure to effective concentrations of repellents, was indistinguishable from theie behavior when both plates were at room temperature or when IR radiation from the warm plate was blocked by covering it with aluminum foil or when the Haller’s organ capsules were occluded with wax. In all of these cases, the ticks stimulated to seek by CO_2_ move at a similar speed—they just do not move preferentially in the direction of the warm plate. Repellents are thought to abolish all host-seeking behavior in ticks, and other cues besides thermal IR might be involved, but these results indicate that one of the strongest of these, CO_2_ from the breath of hosts, is not affected by the repellent concentrations that disrupt thermotaxis. This was confirmed for DEET by showing that ticks exposed to 100 ng•cm^-3^ DEET, twice the rate needed to completely abolish thermotaxis, did not affect the attractiveness of CO_2_. These results suggest that low concentrations of repellents specifically disrupt thermotaxis of questing ticks. Whether they directly affect the thermosensors in the Haller’s organ capsule remains to be seen.

The heat-sensing mechanism of the capsular sensilla remains to be determined. Female *Anopheles gambiae* mosquitos express thermosensitive TRPA1 channels in coeloconic sensilla at the tips of their antennae [21]. These play a critical role in close-range host-seeking, and while they are thought to respond to warm air, their radiant heat sensitivity has not been investigated. However, the capsular structure of coeloconic sensilla indicates that they could be directional IR detectors.

Mosquitoes can detect repellents by olfaction and gustation [29, 30], but it is not clear whether this is related to their repellent mechanism. While it has been shown that citronellal activates TRPA1 channels, this was specifically an isoform involved in olfaction and not the thermosensitive isoform [31, 32]. The potency of citronellal on this channel is quite weak, with EC_50_s of 1.0 and 0.1 mM in *Drosophila* and *Anopheles*, respectively, and the effects of other repellents on it has not been reported.

Previous work on host-sensing in ticks and indeed all ectoparasites must be completely re-evaluated in light of these findings. The thermal IR sense is so exquisitely sensitive and thermotaxic host-seeking behavior so strong that past studies of host-seeking behavior could easily have been inadvertently affected by thermotaxic responses to extraneous heat sources, such as light fixtures or the experimenters themselves.

## Supporting information

S1 Data Raw data files giving the longitudinal positions of all ticks in all thermotaxis trials used in the study. (ZIPX)

S1 Fig Demonstration that a thin, transparent layer of cellophane over aluminum foil, as used in previous attempts to demonstrate heat sensing by ticks [7] and human body lice [17] does not provide an effective radiant heat stimulus. A 1l graduated cylinder filled with water at 37 °C was wrapped partially with aluminum foil and partially with cellophane overlying the upper half of the aluminum foil, giving radiating surfaces of glass, cellophane over glass, cellophane over foil and foil alone. The visible light image is shown on the left and the thermal image is shown on the right, and the table shows the temperatures measured from the thermal image. The cellophane over aluminum foil section, which was intended in the cited studies to provide radiant heat, in fact radiated only enough heat to appear to the thermal imaging camera to be at 24.6 °C.
(TIF)

S2 Fig Y-tube olfactometer (from [15] with permission from Wiley & Sons, Inc.). (TIF)

S3 Fig A. Each graph shows the longitudinal position vs. time of 48 individual *A*. *americanum* females (measured in six groups of eight, with a different line type for each group) after placement in the arena center, at -50 cm, as in Fig 2, with the target plate at 40 °C and located at 0 cm, and the room temperature plate at -100 cm. Horizontal axis units are minutes. Ticks were exposed to the indicated repellent at the indicated concentration before and during the thermotaxis trials. B. Concentration dependence of preference index (PI) and fraction of ticks making a choice of either warm or cold, for DEET, picaridin and 2-undecanone, as indicated. C. Initial speed of female *A*. *americanum* ticks during the first 10 seconds of thermotaxis trials with repellent exposure.
(TIF)

S1 Movie Three thermotaxis bioassay trials, each with 8 unfed virgin adult *A*. *americanum* females, demonstrating thermotaxis and its disruption by DEET. The air circulation tubes can be seen at the top of the arena and are positioned at -10, -50 and -90 cm. The TEC plates are just outside the left and right edges of the video. The dark areas on the left and right ends of the arena are shadows. The counters at each end show the number of ticks within 10 cm of the corresponding TEC plate and correspond to cold plate (left) or warm plate (right) choice scores. Top: Control with both plates at room temperature. Initially, four ticks go right and four go left. Final choice scores are 1 cold plate, 1 warm plate, 6 neither. Middle: warm plate at 37 °C. Five ticks go right initially and final choice scores: 0 cold plate, 5 warm plate, 3 neither. Bottom: warm plate at 40 °C in the presence of 50 ng/cm^3^ DEET. Initially, four ticks go right, three go left and one stays in middle initially but then moves left after a delay. Final choice scores are 1 cold plate, 1 warm plate and 6 neither.
(AVI)

S1 Table Statistical analysis of all thermotaxis data scored as choice tests. (XLSX)
