# Supplementary figures and images for: Ticks home in on body heat: A new understanding of Haller’s organ and repellent action
Source: PLoS One. 2019 Aug 23;14(8):e0221659. doi: 10.1371/journal.pone.0221659 (PMC6707551; doi:10.1371/journal.pone.0221659)

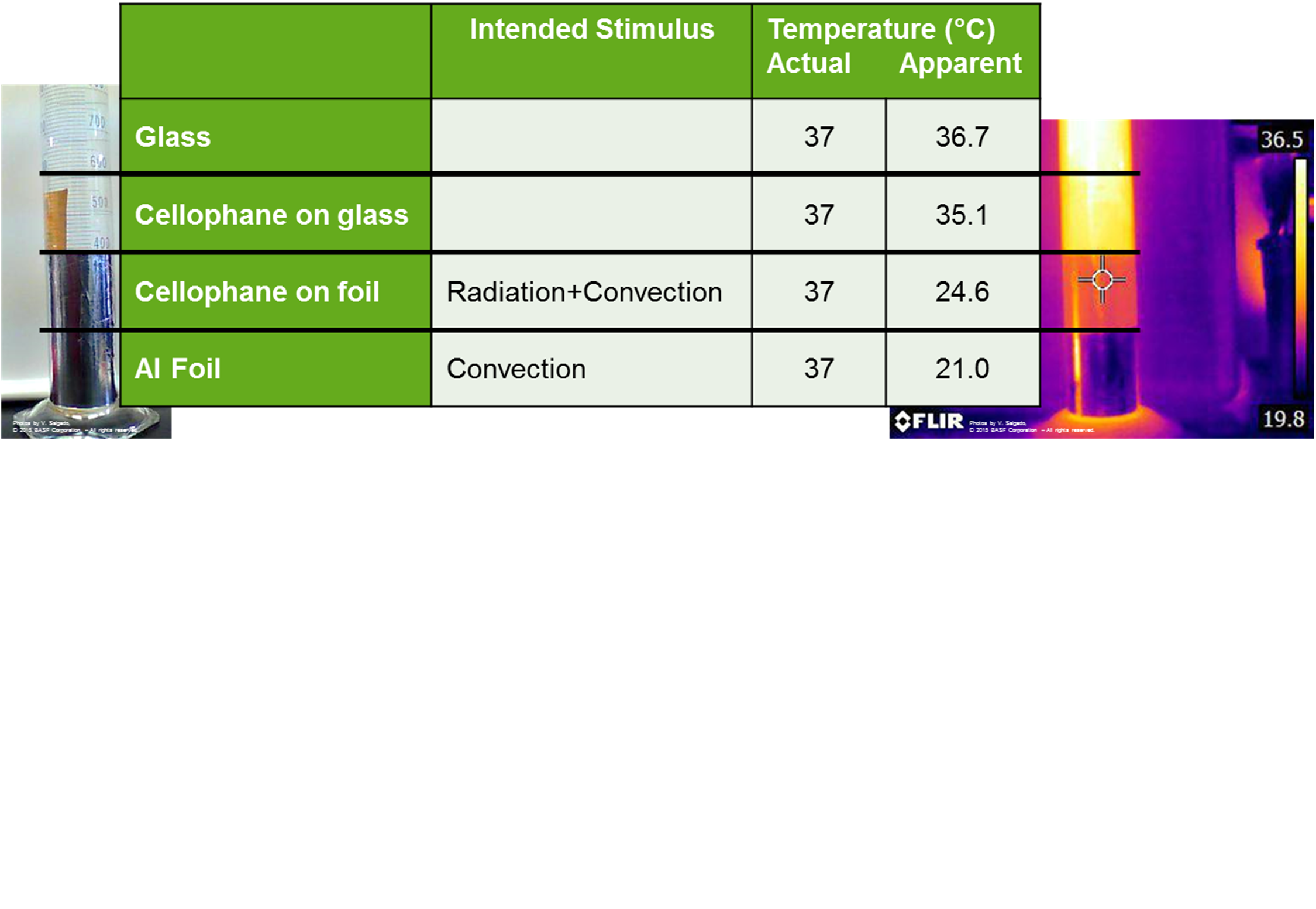

Supplement: S1 Fig — A 1l graduated cylinder filled with water at 37 °C was wrapped partially with aluminum foil and partially with cellophane overlying the upper half of the aluminum foil, giving radiating surfaces of glass, cellophane over glass, cellophane over foil and foil alone. The visible light image is shown on the left and the thermal image is shown on the right, and the table shows the temperatures measured from the thermal image. The cellophane over aluminum foil section, which was intended in the cited studies to provide radiant heat, in fact radiated only enough heat to appear to the thermal imaging camera to be at 24.6 °C. (TIF) [file pone.0221659.s002.TIF]

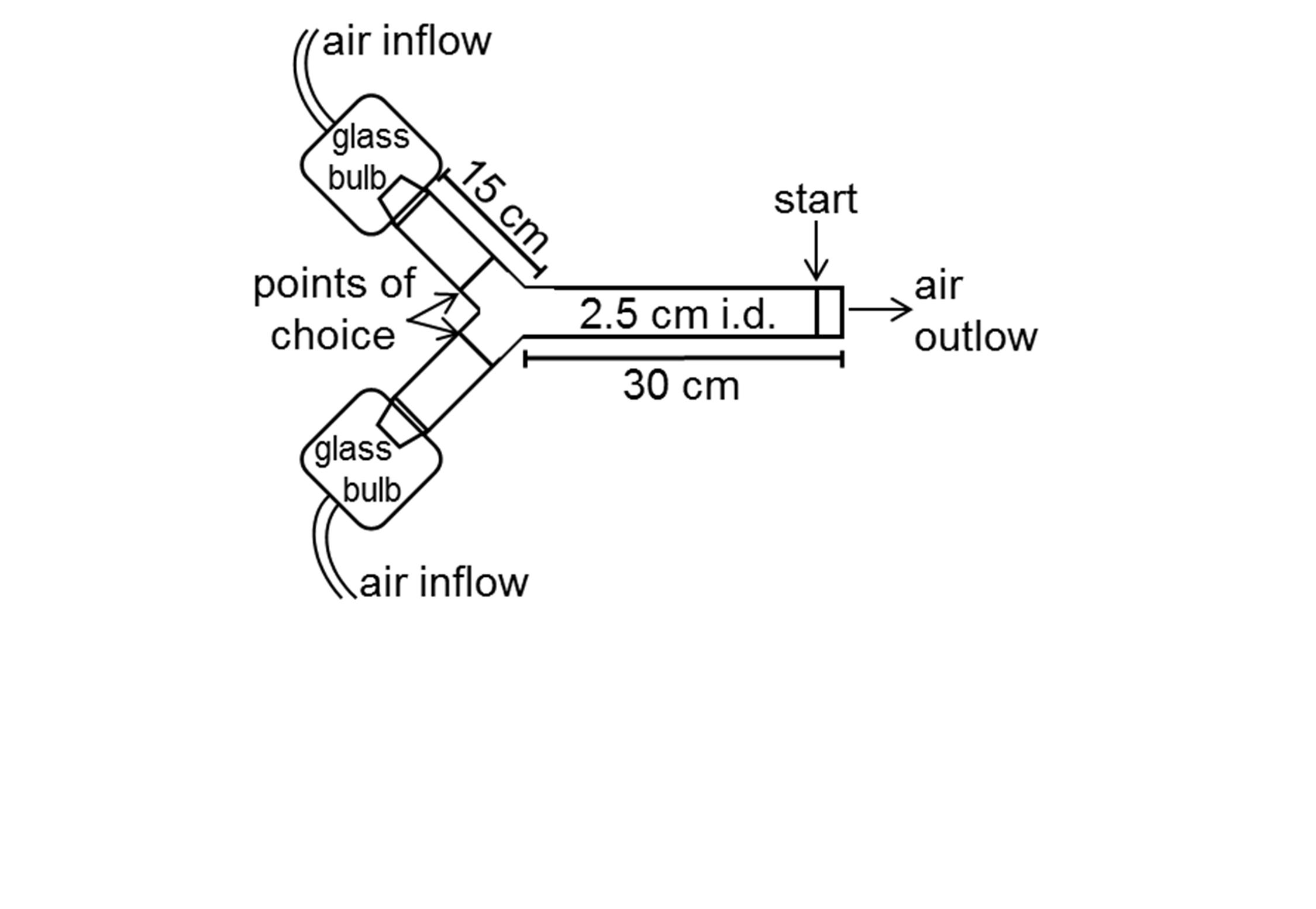

Supplement: S2 Fig — (TIF) [file pone.0221659.s003.TIF]

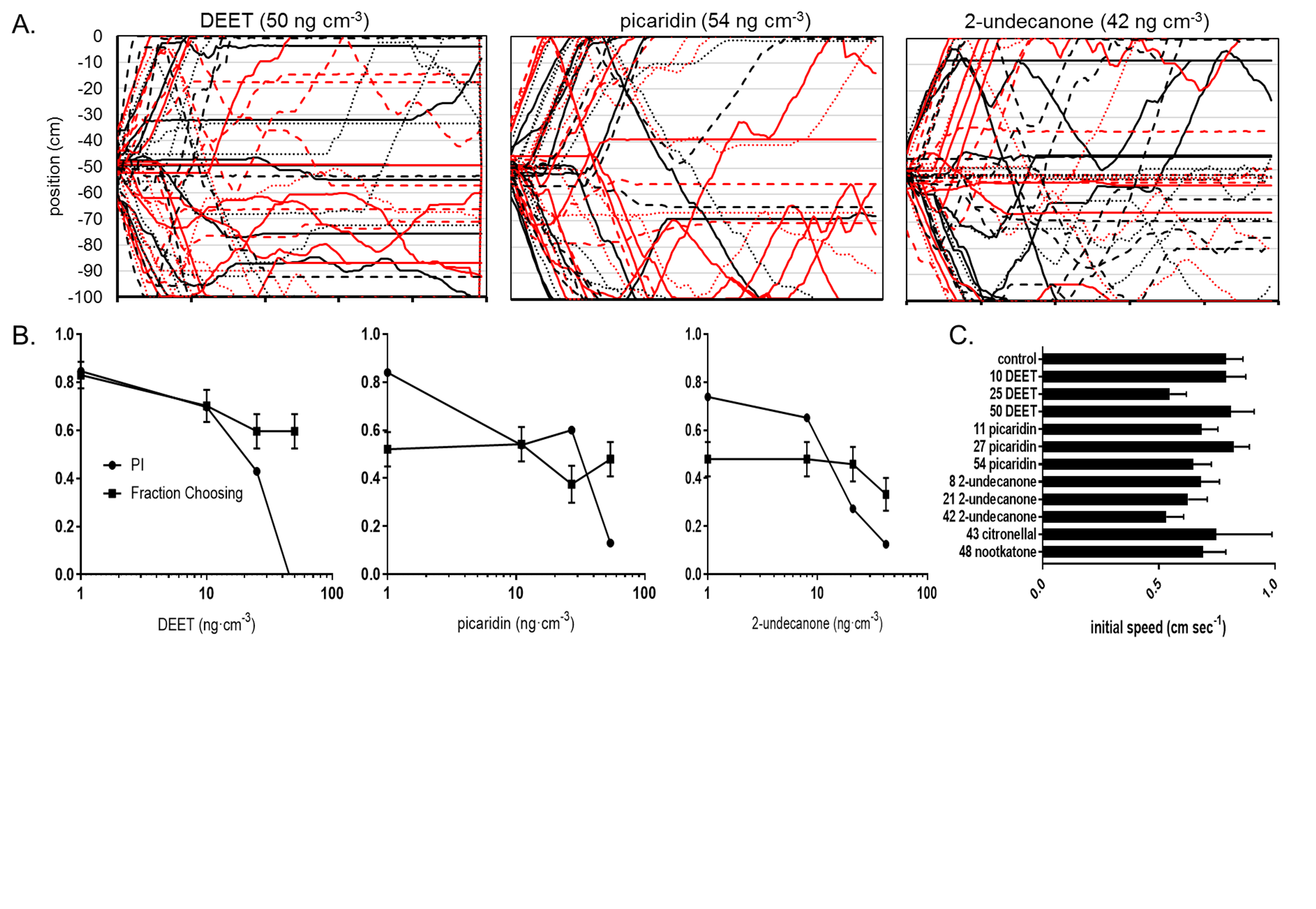

Supplement: S3 Fig — A. Each graph shows the longitudinal position vs. time of 48 individual A. americanum females (measured in six groups of eight, with a different line type for each group) after placement in the arena center, at -50 cm, as in Fig 2, with the target plate at 40 °C and located at 0 cm, and the room temperature plate at -100 cm. Horizontal axis units are minutes. Ticks were exposed to the indicated repellent at the indicated concentration before and during the thermotaxis trials. B. Concentration dependence of preference index (PI) and fraction of ticks making a choice of either warm or cold, for DEET, picaridin and 2-undecanone, as indicated. C. Initial speed of female A. americanum ticks during the first 10 seconds of thermotaxis trials with repellent exposure. (TIF) [file pone.0221659.s004.TIF]
